# Effect of an educational program on mothers' reported practice regarding care of their children with vitamin deficiencies

**DOI:** 10.1186/s40795-026-01253-z

**Published:** 2026-08-11

**Authors:** Eman Abdel Fatah Mousa, Eman Amin Mohamed, Eman Abdel Fatah Ali, Fatma Mohamed Mohamed

**Affiliations:** 1 Faculty of Nursing, Tanta University, Tanta, Egypt; 2 Faculty of Nursing, Ain Shams University, Cairo, Egypt

**Keywords:** Vitamin a deficiency, Vitamin d deficiency, Vitamin c deficiency, Educational program, Mother education, Mothers ' reported practice

## Abstract

**Background:**

Vitamin deficiencies remain significant public health concerns that adversely affect children’s growth and development. Mothers, as primary caregivers, play a key role in shaping their children’s health practices. Therefore, this study aimed to evaluate the effect of an educational program on mothers’ reported practice regarding care of their children with vitamin deficiencies.

**Methods:**

A quasi-experimental study was conducted at the nutrition clinic of the Children’s Hospital, Ain Shams University. A purposive sample of 50 mothers of children diagnosed with vitamin deficiencies (42 vitamin D deficiency, 6 vitamin A deficiency, and 2 vitamin C deficiency) was included. A structured educational program was delivered to mothers, and maternal reported practices were assessed before and after the intervention.

**Results:**

There was a statistically significant improvement (*p* < 0.001) in the total reported practice scores of mothers following the educational program, indicating enhanced care practices related to managing their children’s vitamin needs.

**Conclusion:**

the structured educational program was effectively improved mothers’ reported practices regarding care of their children with vitamin deficiencies, emphasizing the importance of mother education and educational intervention in addressing childhood nutritional deficiencies. Enhancing their knowledge and practices is essential in addressing these nutritional deficiencies. Moreover, the findings of this study could be applied to improve mothers’ roles in the physical, psychological, and social aspects of child care.

**Supplementary Information:**

The online version contains supplementary material available at 10.1186/s40795-026-01253-z.

## Introduction

Children are considered a highly vulnerable population group to nutritional deficiencies due to their rapid growth and continuous development. Adequate nutrition during this stage is important to support normal growth and development. When children is provided with sufficient essential nutrients, particularly vitamins and minerals, theses lead to maintaining child health and ensuring proper growth. Inadequate intake of these nutrients may lead to nutritional deficiencies that negatively affect children’s health and development [1].

Nutritional deficiencies remain a serious global health problem, with a higher burden observed among children living in low- and middle-income countries. These deficiencies are associated with increased rates of childhood illness and death, as many related conditions remain largely preventable through appropriate nutritional interventions. Deficiencies of essential important vitamins, particularly vitamins A, D, and C, are widely reported among children and are linked to adverse health consequences. Although substantial global efforts have been made to address these issues, vitamin deficiencies remain highly prevalent in resource-limited regions such as Southeast Asia, Sub-Saharan Africa, and the Middle East [2].

Many studies have stressed that mothers factors and socioeconomic conditions have an effect on children’s nutritional status. Under nutrition has been reported to be more prevalent among children from rural areas compared to those from urban settings [2, 3]. In Egypt, disparities in socioeconomic status have been related to higher rates of childhood under nutrition [4, 5]. Evidence from similar studies suggests that giving mothers educational program may have positive effect on mothers’ nutritional knowledge, and their practices, regarding care of their children with vitamin deficiencies [6].

The presence of vitamin deficiencies in children is affected by a variety of factors, including poor dietary habits, limited mothers’ knowledge about nutrition, socioeconomic challenges, and reduced access to healthcare services. To decrease these deficiencies, targeted interventions are necessary. In particular, educational programs designed for mothers play an important role in enhancing their practices, which in turn positively influence children’s dietary behaviors, growth and development [7].

Mothers play multiple crucial roles in the care of their children. They are not only primary caregivers, but also serve as counselors and educators, guiding children’s behaviors and choices. These roles are particularly important when it comes to nutrition, as mothers’ guidance, counseling, teaching and practices directly affect children’s eating habits. Ensuring that mothers are well-informed about healthy nutrition, is therefore essential for supporting optimal growth and development in children [8, 9].

### Significance of the study

Vitamin deficiencies in children represent a persistent and under recognized public health issue with profound effects on growth, development, and survival. Globally, under nutrition accounts for nearly 50% of deaths among children under five, with the greatest impact in low- and middle-income regions. In the Middle East and North Africa (MENA), the prevalence of vitamin D deficiency ranges between 20 and 80% in apparently healthy children, despite abundant sunlight—highlighting a paradox driven by cultural, environmental, and lifestyle factors [10–12].

In Egypt specifically, recent evidence confirms high rates of vitamin D deficiency among healthy children, underscoring the need for local attention. Vitamin A deficiency remains the world’s leading preventable cause of childhood blindness and compound mortality through infections, An estimated 250 000–500 000 children who are vitamin A-deficient become blind every year, and half of them die within 12 months of losing their sight. Deficiency of vitamin A is associated with significant morbidity and mortality from common childhood infections, and is the world’s leading preventable cause of childhood blindness [13]; while vitamin C deficiency compromises immune defense and increases susceptibility to respiratory illnesses [14].

#### Aim of the study

This study aimed to evaluate effect of educational program on mothers’ reported practice regarding care of their children with vitamins deficiency through the following:

- Assessing mothers’ level of knowledge regarding care of their children with vitamin deficiency through program phases.
- Assessing mothers’ level of reported practice regarding care of their children with vitamin deficiency through program phases.
- Developing and implementing educational program for mothers based on needs assessments about vitamins deficiency through program phases.
- Evaluating effect of educational program on mothers’ reported practice regarding care of their children with vitamin deficiency through program phases.

### Research hypothesis

Educational program improved and had positive effect on mothers’ reported practice regarding care of their Children with vitamins deficiency.

## Subjects and methods

### Research design

A Quasi-experimental was used in the study.

### Setting

The study was conducted at nutritional clinic of the Children Hospital affiliated to Ain Shams University.

### Participants

A purposive sample of children aged 2–12 years diagnosed with vitamin A, D, or C deficiencies was recruited. Children with chronic illnesses were excluded to maintain comparability. The primary unit of analysis was the mothers, who shared inclusion characteristics: recruited from the same setting, having a child with vitamin deficiency, and participated in a the same educational program. This ensured sufficient homogeneity for assessing mothers’ reported practices.

### Sample size

The sample size was calculated using the formula described by Charan& Biswas [15]:

$$\:\boldsymbol{\text n}\frac{{\boldsymbol{t}}^{2\times\:\boldsymbol{p}(1-\boldsymbol{p})}}{\boldsymbol{m}2}$$

where n is the required sample size, t is the standard normal deviate at 95% confidence level (1.96), p is the estimated proportion of vitamin deficiency in children (0.034), and m is the margin of error (0.05).

The required sample size was therefore approximately 50 mothers, which was adopted in this study.

### Sample type

Purposive sampling.

### Inclusion and exclusion criteria for children

Children aged between 2 and 12 years who had been clinically diagnosed with vitamin A, D, or C deficiency were eligible to participate. Children with any chronic illness were excluded from the study.

### Inclusion and exclusion criteria for mothers

Eligible participants were mothers of children diagnosed with vitamin deficiencies (A, D, or C) who agreed to participate in the study. Mothers who refused participation were excluded.

### Tools of data collection

The data for this study were collected using two main instruments, each clearly structured into distinct sections:

First tool, a pre-designed questionnaire sheet was utilized, comprising three sections. The first section addressed the characteristics of both mothers and children. It included 11 items: five items focused on mothers’ characteristics (e.g., age, education, residence, occupation, and family income) and six items on children’s characteristics (e.g., age, gender, birth order, educational level, diagnosis, and age at onset of the condition). An English version of this section is available as Supplementary File 1.

The second section of the questionnaire assessed mothers’ knowledge regarding care of children with vitamin deficiencies. Adapted from Xu et al. [16], Abbas et al. [17], and Marks [18].

In the third section, clinical data assessment. This section concerned with knowledge related to child nutritional history, physical examination and anthropometric measurement (BMI) (adapted from Liu et al. [19], and the food behavior checklist (adapted from Ko MM et al. [20], assessing child eating behaviors.

Second tool, Vitamin-Related Reported Practice Questionnaire (pre–post test) was administered to assess mothers’ reported practices regarding care of their children with vitamin deficiencies. This tool composed of three parts: first, vitamin A–related reported practices adapted from Xu et al. [16], These assessed mothers’ reported practices regarding care of their children with vitamin A deficiency .

Second, vitamin D–related reported practices, adapted from Amiri et al. [21], These assessed mothers’ reported practices regarding care of their children with vitamin D deficiency .

Third, vitamin C–related reported practices, adapted from Marks [18] and Hahn et al. [22]. These assessed mothers’ reported practices regarding care of their children with vitamin C deficiency.

### Scoring system for mothers’ reported practice

A three-point response scale was applied to each item (always = 2, sometimes = 1, never = 0). Total scores were calculated and interpreted as adequate reported practice (≥ 70% of the maximum possible score) or inadequate reported practice (< 70%).

### Ethical considerations

The study was approved by Scientific Research Ethics committee of the Faculty of Nursing, Ain Shams University before starting the study(Approval No: [25.08.822], Date: [14/7/2025]. Written informed consent was obtained from all participating mothers prior to data collection.

### Administrative design

An official approval to conduct this was obtained from the Dean of the Faculty of Nursing at Ain Shams University; a letter containing the title and aim was directed to the director of Ain Shams pediatric Hospital; and oral approval from the director was required to obtain this approval for data collection.

### Statistical design

Data were entered and analyzed using SPSS (version 22). Graphics were done using Excel. Quantitative data were presented as mean (X̅) and standard deviation (SD). Qualitative data were presented in the form of frequency distribution tables, numbers, and percentages. The Chi-square (χ²) test was applied to compare pre- and post-program results, and McNemar test was used to assess changes in paired categorical data. Baseline homogeneity of participants’ characteristics was assessed using Chi-square tests to ensure comparability of groups before program implementation. The level of significance was set at p-value < 0.05 for all statistical tests.

### Reliability of the tool

The reliability of the proposed tools was tested using Cronbach’s alpha through the SPSS software package. The value was 0.87 for the Vitamin-Related Reported Practice Questionnaire, indicating that the tool was reliable for detecting and measuring the objectives of the study.

### Pilot study

It was carried out on 10% of the studied mothers (5 mothers) to evaluate the applicability of the study tools and estimate the proper time required for answering the required data. All participants in the pilot study were included in the main study while, no modifications were made to the tools of data collection.

### Field work

#### Assessment phase

The assessment phase was conducted by the researcher for all study subjects. Mothers who met the inclusion criteria were approached, and the researcher explained the purpose and expected duration of the educational program to gain their cooperation. Oral approval to participate was obtained from each mother prior to data collection.

#### Planning phase

Based on the identified needs of the mothers, the researcher developed an educational program designed to address knowledge and practice gaps. Supporting teaching materials were also prepared to facilitate both the theoretical and practical components of the sessions.

#### Implementation phase

The implementation phase extended over six months, from May to October 2024. The researcher was available during morning shifts twice weekly (Sundays and Wednesdays). At the outset, the researcher introduced herself to the participating mothers, explained the study objectives, and highlighted the expected impact of the program on their reported practices regarding care of their children with vitamin deficiencies. Written informed consent was obtained from each mother prior to data collection.

Baseline data were collected before the educational program through administration of the structured questionnaire and the mothers’ reported practice assessment sheet. Each mother required approximately 30 min to complete the questionnaires, and for illiterate participants the researcher conducted face-to-face interviews and filled in the responses on their behalf.

The educational program was delivered in small groups of four to six mothers across six structured sessions that covered both theoretical and practical content. Each session lasted 30 to 45 min and began with a summary and feedback on the previous session. Simple Arabic language was used to match the mothers’ level of understanding. At the end of each session, mothers’ inquiries were addressed to clarify any misconceptions. A variety of instructional methods were employed, including lectures and group discussions, supported by visual aids such as pictures, colored booklets, and posters.

#### Educational program

The educational program was developed by the researcher based on mothers’ needs assessment and a review of the related literature. It addressed both theoretical and practical aspects of vitamin deficiencies (A, D, and C) and consisted of six structured sessions.

#### General sessions (four sessions)

All participating mothers attended four general sessions regardless of the type of vitamin deficiency in their children. Two theoretical sessions focused on basic nutrition concepts, food groups and their importance, types and functions of vitamins, and under nutrition (definition, causes, manifestations, and its impact on child health, growth, and development). Two practical sessions trained mothers on healthy food habits, meal and snack planning, food hygiene and safety, safe cooking and storage practices to preserve nutrients, infection prevention, and encouraging children’s physical activity.

#### Vitamin-specific sessions (two sessions)

“In addition to the general sessions, mothers attended two sessions tailored to their child’s specific vitamin deficiency: one theoretical session and one practical session.” For vitamin A deficiency, the theoretical session covered causes, manifestations, and complications, while the practical session focused on preparing vitamin A–rich foods, supplementation, vaccination adherence, and avoiding factors that reduce absorption of vitamin A.

For vitamin D deficiency, the theoretical session addressed importance, causes, manifestations and complication of deficiency, while the practical session emphasized safe sun exposure, dietary sources, supplementation, and avoiding factors that affect synthesis and absorption of vitamin D. For vitamin C deficiency, the theoretical session explained causes, symptoms, and consequences, while the practical session trained mothers on preparing vitamin C–rich foods, supplementation, and healthy cooking methods that preserve vitamin **C.**

#### Evaluation phase

The evaluation phase aimed to assess the effect of the educational program on mothers’ reported practices regarding the care of their children with vitamin deficiencies. Mothers’ reported practices were measured before and after program implementation and post-test results were compared with pre-test findings to determine the effectiveness of the program.

### Strengths of the study

This study addressed an important public health concern, namely vitamin deficiencies in children, which have both short- and long-term consequences for child development and wellbeing. By focusing on mothers as the primary caregivers, the intervention enhanced the relevance and applicability of the educational program to real-life settings. The study employed an educational intervention rather than an observational approach, which allowed for practical application and the potential to promote behavioral change. The use of a pre-test/post-test design enabled the direct evaluation of the program’s effect on mothers’ practices. Furthermore, the educational content was tailored to the local context, thereby increasing the likelihood of acceptance and comprehension by the participating mothers.

### Limitations of the study

The findings of this study should be interpreted in light of certain limitations. The limited geographic and demographic scope of the sample may restrict the generalizability of the results. In addition, reliance on mother-reported practices could have introduced reporting bias or social desirability bias. The short follow-up period, with assessment conducted shortly after the intervention, may limit conclusions regarding long-term behavioral change.

## Results

### Analysis of the results

#### Socio-demographic characteristics of the study subjects

As shown in Table 1, 42% of the studied mothers were in the age category 25–<30 years, 60% lived in rural areas, 60% had secondary school education, and 60% were not working. In addition, 72% did not have sufficient monthly income. The distribution of socio-demographic characteristics demonstrated relative homogeneity, with no substantial variations across groups (*p* > 0.05), ensuring comparability of participants.

**Table 1:** Distribution of the studied mothers according to their characteristics (*N*=50)

Characteristics of mothers	No	%	X2	*p*
Mother’s age			2.1	0.15
20-<25	6	12		
25-<30	21	**42**		
30-<35	21	42		
35-<40	2	4		
Mean ± SD 29.4 ± 3.73				
Residence			**1.59**	**0.20**
Urban	20	**40**		
Rural	30	60		
Level of education			**4.1**	**0.05**
illiterate	2	4		
Read and write	6	12		
Secondary school(intermediate education)	30	60		
High school or (university)education	12	24		
Occupation			**2.47**	**0.11**
Working	20	40		
Not working/housewife	30	**60**		
Monthly income			**3.1**	**0.07**
Enough	14	28		
Not enough	36	**72**		

The distribution of mothers’ socio-demographic characteristics demonstrated relative homogeneity, with no substantial variations across groups

Regarding the children characteristics (Table 2), most children were preschoolers (70%), and vitamin D deficiency was the most common diagnosis (84%). The distribution of children’s socio-demographic and clinical characteristics demonstrated relative homogeneity, with no statistically significant differences detected regarding child’s age group (χ² = 0.29, *p* = 0.59), gender (χ² = 3.9, *p* = 0.05), child ranking (χ² = 1.6, *p* = 0.20), level of education (χ² = 3.5, *p* = 0.05), or diagnosis type (χ² = 2.0, *p* = 0.16). Regarding the age at disease discovery, a statistically significant difference was observed (χ² = 19.8, *p* = 0.00005), indicating that most children were diagnosed at earlier ages (1–<6 years), while very few were diagnosed later (6–12 years).

**Table 2:** Distribution of the studied children according to their characteristics (*N*=50)

Characteristics of the children	No	%	X2	*p*
Child’s age (in years)			0.29	0.59
2-<6	36	**72**		
6-< 9	6	12		
9 < 12	8	16		
Mean ± SD5.46 ± 2.47				
Gender			**3.9**	**0.05**
Male	32	**64**		
Female	18	36		
Child ranking			**1.6**	**0.20**
1st	24	**48**		
2nd	16	32		
3rd	7	14		
4th and More	3	6		
Level of education			**3.5**	**0.05**
Preschool	35	**70**		
Primary school	13	26		
Secondary school	2	4		
Child diagnosis			**2.0**	**0.16**
Vitamin A deficiency	6	12		
Vitamin D deficiency	42	**84**		
Vitamin C deficiency	2	4		
Child age When discovering the disease			**19.8**	**0.00**
1-<3 year	26	**52**		
3-<6 years	22	44		
6–12 years	2	4		

Most children were preschoolers (70%), and vitamin D deficiency was the most common diagnosis (84%). A statistically significant difference was observed only regarding the age at disease discovery (*p* <0.001)

#### Participant recruitment

**Fig. 1:**
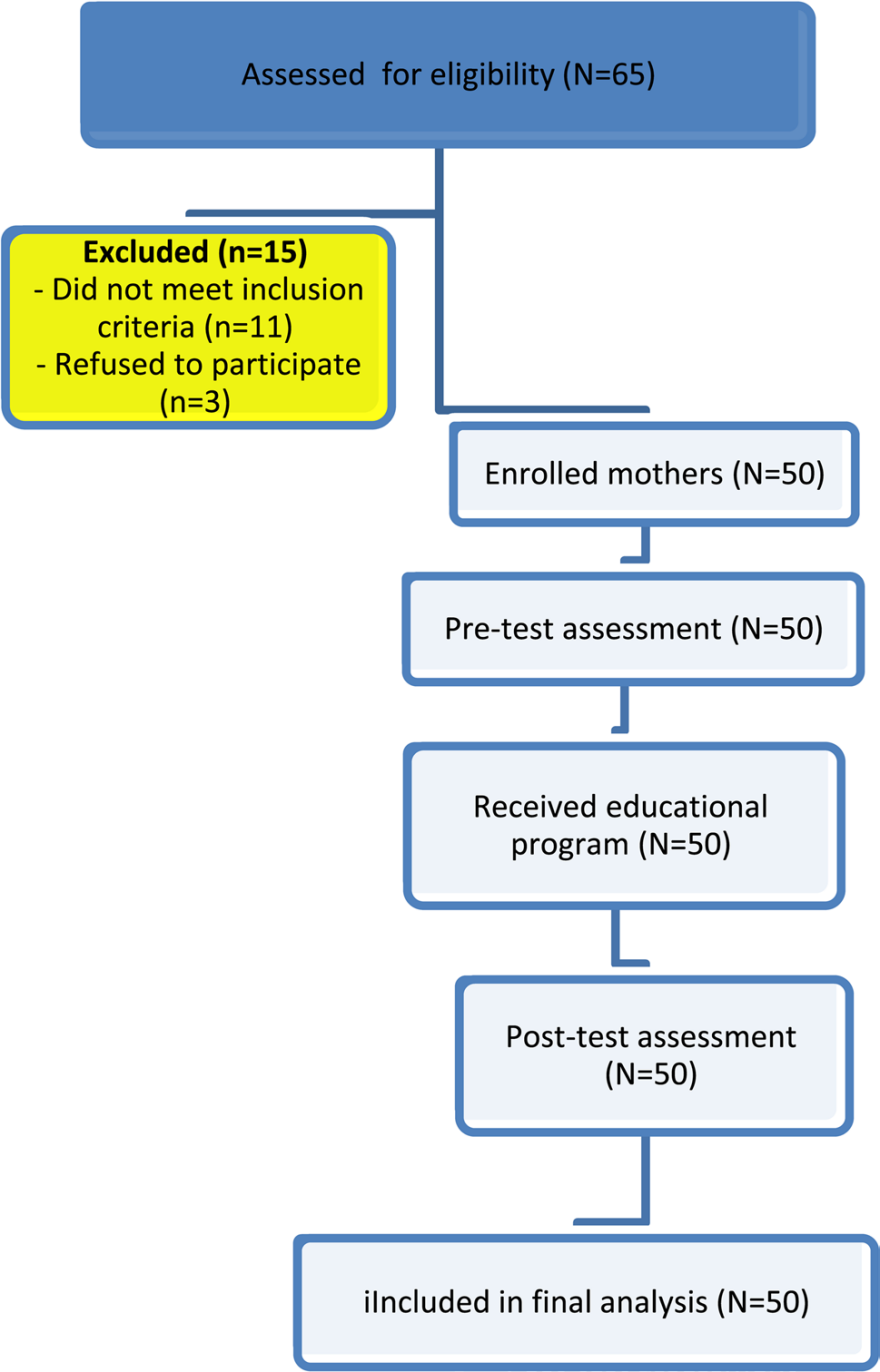
Flow chart of participants’ recruitment

As illustrated in Fig. 1, a total of 65 mothers were assessed for eligibility, of whom 15 were excluded. Finally, 50 mothers were enrolled, completed the intervention, and were included in the final analysis.

#### Pre-program knowledge assessment

Table 3 illustrates that most mothers had low baseline knowledge regarding vitamin deficiency. This assessment guided the content and structure of the educational program, ensuring it addressed mothers’ learning needs. Subsequently, the program led to a statistically significant improvement in mothers’ reported practice scores (*p* < 0.001, η² = 0.64).

**Table 3:** Distribution of the studied mothers’ level of total score of knowledge, regarding Vitamin deficiencies (A,D & C) before implementation of the educational program (*N*=50)

Level of total knowledge	Mother’ level of total Knowledge	
	Pre-Test	
	No	%
Satisfactory	3	6
Unsatisfactory	47	**94**

Most mothers (94%) had unsatisfactory knowledge regarding vitamin deficiency before the educational program

#### Post-program practice improvement

Table 4 illustrates that There was a statistically significant improvement in mothers’ total practice scores after implementation of the educational program. McNemar test showed a significant difference (*p* < 0.001), with a large effect size (η² = 0.64), indicating substantial improvement in mothers’ practices between pre- and post-program phases.

**Table 4:** Distribution of the studied mothers’ level of total score of practice regarding Vitamin deficiencies (A,D & C) between pre-program and post-program (*N*=50)

Level of total practice	Mother’ level of total practice				Mc Nemar test	*P*-value
	Pre-Test		Post-Test			
	No	%	No	%		
Adequate	11	22	35	**70**	20.1	*P* < 0.001
Inadequate	39	78	15	30		
Effect size	0.64					

Mothers’ total practice scores significantly improved after the educational program (*p* <0.001), with a large effect size (η²= 0.64), indicating substantial enhancement in mothers’ practices

### Correlation analysis

Additionally, correlation analysis was conducted as an exploratory step to examine possible associations between mothers’ socio demographic characteristics and their practice. The distribution of mothers’ socio-demographic characteristics demonstrated relative homogeneity. No statistically significant differences were found regarding mothers’ age (χ² = 2.1, *p* = 0.15), residence (χ² = 1.59, *p* = 0.20), level of education (χ² = 4.1, *p* = 0.05), occupation (χ² = 2.47, *p* = 0.11), or monthly income (χ² = 3.1, *p* = 0.07).

## Discussion

This study highlights the persistence of vitamin deficiencies as an important health issue that negatively impacts child growth and development. The findings of the present study are consistent with previous evidence presented in the introduction, which emphasized the adverse effects of inadequate nutrition on child health and development [1, 14]. These results further support global reports on the high prevalence and consequences of vitamin deficiencies in children [23, 24]. Similarly, Haldar et al. [7] highlighted the multifactorial nature of childhood malnutrition, while Hegde et al. [8] reported that nutritional awareness programs among mothers had a significant impact on improving children’s dietary practices, supporting the effectiveness of the current educational intervention.

Regarding characteristics of the studied mothers, the finding of this study showed that, more than half of studied mothers live in rural area. This finding was in accordance with Yunitasari et al., [3] who indicated that malnourished children are more common in rural than in urban areas.

Additionally, this finding was in consistence with Pal et al. [2]. Also indicated that under nutrition was more common in children from rural regions than in children from urban areas. These could be related to a lack of health knowledge, and dietary awareness among rural mothers.

Also, the finding revealed that less than three quarters don’t have enough monthly income, this finding was in accordance with Hany et al. [4] whose study aimed to assess the socioeconomic-related risk factors that contribute to the occurrence of malnutrition among the under-five children and discovered that malnutrition state was more common among the lower social class.

Furthermore, the current study clarified that less than two third of studied mothers had secondary school education (intermediate education). These explain that mother education important factors in determining nutritional status of child because it reflects on knowledge and practice of mothers toward caring of their children.

These findings were in an agreement with results of Aboakrab [5], who conducted study to determine factors associated with inequality in under nutrition among Egyptian children and concluded that mothers’ education contributed to socioeconomic disparities in childhood under nutrition.

From the researcher point of view, mother characteristic is important factors in determining level of knowledge that mother with intermediate education has low level of knowledge regarding child health. Mother who lives in rural area had children with nutritional deficiency due to limited access for health facility.

With regard to the characteristics of the studied children, the present study showed that males constituted less than two thirds of the study sample. This finding is comparable to results reported by Ahad et al. [12] who examined the risk factors of vitamin D deficiency among school-aged children in District Peshawar. Their study explored that there is a higher prevalence of vitamin D deficiency and insufficiency among male children, whereas lower rates were found among females.

Moreover, the finding of this study revealed that, the majority of studied children had vitamin D deficiency diagnosis. These may be due to not eating food rich in vitamin D and not exposed to sun regularly. This finding was in agreement with Gad et al., [10], Zeinab et al., [11] whose study was conducted to assess vitamin-D status among children and adolescents, and to find out predictors for its deficiency/ insufficiency among the studied groups and revealed that there is a high prevalence of VDD as the majority of studied sample were below 30ng/dl. Children showed more than half of studied children were deficient and severe deficient and less than one third of studied children were vitamin D insufficiencies.

The relative homogeneity of the sample at the mothers’ level supports the validity of the observed improvement in reported practices, while age variability among children did not affect the primary outcomes.

The findings of the present study, which revealed that more than half of the studied mothers had insufficient baseline knowledge regarding vitamin deficiencies, are consistent with previous evidence from the Middle East. For instance, Bassam and Abd-Elmageed [9] reported that the majority of mothers in the Qassim region of Saudi Arabia demonstrated inadequate knowledge, practices, and attitudes toward vitamin D deficiency in their children.

The current study demonstrated that an adequate level of reported practice was observed in a small proportion of mothers only before implementation of educational program, while a notable increase in proportion of mothers’ reported practice was evident after program implementation. This improvement is in line with findings reported by Elnagar et al. [6], who documented marked enhancements in mothers’ practices after receiving education based on the Health Belief Model. The large effect size observed in the present study (η² = 0.64) indicates that the educational program had a meaningful and practically significant impact, suggesting that structured, educational education can effectively improve mothers’ practices regarding vitamin deficiency .

The researcher believes that the observed improvement in mothers’ reported practices following the implementation of the educational program reflects the effectiveness of the health education strategies used. This improvement can be attributed to the program’s well-structured content, which was tailored to match the mothers’ educational and cultural backgrounds and delivered in a clear, engaging manner.

Active participation during the educational sessions-through open discussion and interaction-played a significant role in correcting misconceptions and reinforcing appropriate practices.

The researcher considers this result as strong evidence of the importance of community health education in improving child and family health outcomes. It further highlights the role of health education as an essential component of primary healthcare service.

## Conclusion

The educational program effectively enhanced mothers’ reported practice, addressing the study’s main aim.The findings of this study can be applied beyond nutritional practices, contributing to improvements in children’s physical growth, psychological well-being, and social development through better maternal care and awareness.

## Recommendations

This study highlights the need for context-specific interventions to address vitamin deficiencies among children. Regular maternal nutrition education sessions should be implemented within primary healthcare settings and integrated into routine antenatal and postnatal services to ensure wide coverage. Low-cost educational materials and digital tools such as SMS reminders and mobile applications can be used to reinforce key messages. Community health workers should be empowered to deliver home-based counseling, particularly in rural areas with limited healthcare access. Strengthening the capacity of healthcare providers and fostering collaboration with schools and local organizations are essential to sustain improvements in Mothers knowledge and practices.

## Supplementary Information

Supplementary Material 1.

## Data Availability

The datasets used and/or analyzed during the current study are available from the corresponding author on reasonable request.
