# Supplementary material for: Effect of an educational program on mothers' reported practice regarding care of their children with vitamin deficiencies
Source: BMC Nutr. 2026 Aug 11;12:156. doi: 10.1186/s40795-026-01253-z (PMC13459633; doi:10.1186/s40795-026-01253-z)
Supplement: Supplementary file 1 [file 40795_2026_1253_MOESM1_ESM.pdf]

Survey Form to Measure Effect of an Educational Program on Mothers ' Reported Practice Regarding Care of Their Children with Vitamin Deficiencies

Supplementary File 1: Part I of the Questionnaire (English Version)

Part I: Characteristics of study subjects:

A: Characteristics of Mothers:

1. **Mother's Age(in years)**
   ☐ 20 – <25 years
   ☐ 25 – <30 years
   ☐ 30 – <35 years
   ☐ 35 – <40 years
   ☐ 40 years or more
2. **Residence**
   ☐ Urban
   ☐ Rural
3. **Level of Education**
   ☐ Illiterate
   ☐ Can read and write
   ☐ Secondary school (intermediate education)
   ☐ High school or university education
4. **Occupation**
   ☐ Working
   ☐ Not working / Housewife
5. **Monthly Income**
   ☐ Enough
   ☐ Not enough

B: Characteristics of the Children:

1. **Child's Age (in years)**
   ☐ 2 – <6
   ☐ 6 – <9
   ☐ 9 – 12

2. **Gender**
   ☐ Male
   ☐ Female
3. **Child Ranking in the Family**
   ☐ 1st
   ☐ 2nd
   ☐ 3rd
   ☐ 4th or more
4. **Level of Education**
   ☐ Preschool
   ☐ Primary school
   ☐ Secondary school
5. **Child's Diagnosis**
   ☐ Vitamin A deficiency
   ☐ Vitamin D deficiency
   ☐ Vitamin C deficiency
6. **Child's Age (in years)When the Deficiency Was Discovered**
   ☐ 1 – <3 years
   ☐ 3 – <6 years
   ☐ 6 – 12 years
